# Comparative analysis of robot-assisted and open approach for PSMA-radioguided surgery in recurrent prostate cancer

**DOI:** 10.1007/s00259-023-06460-5

**Published:** 2023-10-13

**Authors:** Francesca Ambrosini, Fabian Falkenbach, Lars Budäus, Thomas Steuber, Markus Graefen, Daniel Koehler, Sophie Knipper, Tobias Maurer

**Affiliations:** 1 Martini-Klinik Prostate Cancer Center, University Hospital Hamburg-Eppendorf, Martinistraße 52, 20246 Hamburg, Germany; 2 IRCCS Ospedale Policlinico San Martino, Genoa, Italy; 3 Department of Urology, University Hospital Hamburg-Eppendorf, Hamburg, Germany; 4 Department of Diagnostic and Interventional Radiology and Nuclear Medicine, University Hospital Hamburg-Eppendorf, Martinistraße 52, 20246 Hamburg, Germany

**Keywords:** Prostate cancer, Prostate-specific membrane antigen–radioguided surgery, Salvage lymph node dissection

## Abstract

**Purpose:**

To compare the oncological and surgical outcomes of patients with recurrent prostate cancer (PCa) who underwent either open or newly established robot-assisted salvage prostate-specific membrane antigen–radioguided surgery (PSMA-RGS).

**Materials and methods:**

Patients who consecutively underwent PSMA-RGS for PCa recurrence between January 2021 and December 2022 were identified. The rate of complete biochemical response, biochemical recurrence-free survival [BFS], and the rate of salvage therapy were evaluated. Univariable and multivariable regression models tested the association between the surgical approach and surgical outcomes.

**Results:**

Overall, 85 patients were selected, with 61 patients (72%) undergoing open PSMA-RGS and 24 patients (28%) receiving a robot-assisted approach. The oncological outcomes of the two groups were comparable (12-month BFS: 41% (Confidence interval (CI): 29–58%) vs. 39% (CI: 19–79%), *p* = 0.9, respectively). According to multivariable regression models, the robotic approach did not significantly influence estimated blood loss (EBL) (*β* = −40, 95% CI: −103, 22; *p* = 0.2) and significantly increased operative time (OT) (*β* = 28, 95% CI: 10, 46; *p* = 0.002). No Clavien-Dindo III–V complications were reported in the robotic group.

**Conclusion:**

Both, the open as well as the robot-assisted approach for PSMA-RGS had comparable oncological outcomes. No safety concerns arose for the robotic-assisted approach offering a potentially improved quality of life for patients.

## Introduction

In recent years, the use of positron emission tomography (PET) agents targeting prostate-specific membrane antigen (PSMA) has greatly improved the accuracy of prostate cancer (PCa) metastasis detection compared with conventional imaging [1, 2]. This advance has led to a shift in treatment approaches from systemic therapy to salvage metastasis-directed therapy (MDT) for PCa recurrence [3–5]. PSMA-radioguided surgery (PSMA-RGS) has emerged as a surgical strategy to improve intraoperative localization of PCa recurrence, benefiting from the binding of radioligand to PSMA-positive PCa lesions [6–8].

The oncological outcomes of salvage PSMA-RGS are promising. Reportedly, up to 32% of patients are free of biochemical recurrence (BCR) after 2 years without the need for further treatment [9]. Currently, open surgical procedures are most commonly used at PSMA-RGS and have been shown to be very effective in lymph node dissection [6, 9, 10] and resection of local recurrence [9, 11]. In recent decades, minimally invasive surgery has gained popularity in surgery, but its role in PSMA-RGS is still relatively unexplored. The first clinical cases of robot-assisted salvage PSMA-RGS of lymphatic metastases were described in 2019 [12].

To date, only one prospective feasibility study demonstrated the safety and feasibility of robot-assisted PSMA-RGS and encouraged further prospective trials [13].

In this study, we assessed the feasibility of robot-assisted PSMA-RGS, evaluated the agreement between ^99m^Tc-PSMA-I&S enhancement and histopathology, and analyzed short-term oncological outcomes [13]. Despite the valuable insights gained from these existing data, no studies to date have compared outcomes between open and robot-assisted salvage PSMA-RGS.

We present the results of a comparative analysis that aimed to evaluate the oncological and surgical outcomes of patients with recurrent prostate cancer (PCa) who underwent either open or robot-assisted PSMA-RGS.

## Materials and methods

### Study population

**Fig. 1:**
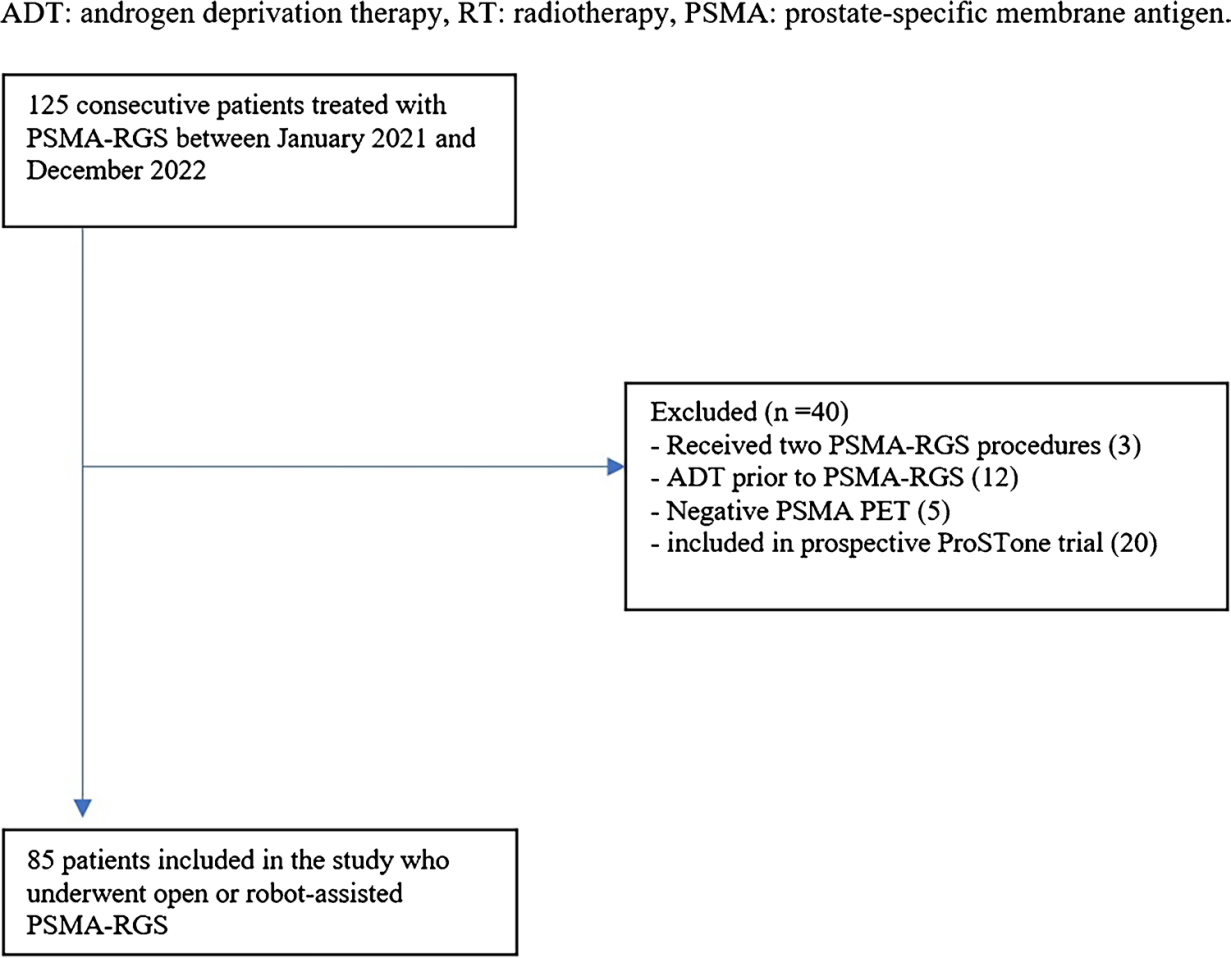
Cohort flow diagram of patients included in the study

Patients who consecutively underwent PSMA-radioguided surgery (PSMA-RGS) for PCa recurrence at one tertiary referral center between January 2021 (when the robotic PSMA-RGS program began) and December 2022 were selected from our prospectively collected institutional review board-approved database (institutional review board of Hamburg (2019-PS-09; PV7316), Germany). Patients had at least one positive lesion detected on PSMA PET imaging in the pelvis or retroperitoneum suspicious for lymph node metastases (LNMs) or local recurrence. Patients were excluded if they had received androgen deprivation therapy (ADT) within 6 months prior to PSMA PET, negative PSMA PET, secondary PSMA-RGS, primary treatment different from RP, missing follow up or enrollment in the prospective ProSTone trial (NCT04271579) (Fig. 1) [14].

All surgical procedures were performed by a single highly experienced surgeon, who had performed more than 800 open RPs, more than 200 robot-assisted RPs, and >250 open PSMA-RGS. Given the initially limited experience, the robotic approach was performed in cases involving unilateral positive pelvic lesions or retrovesical/paravesical lesions detected by PSMA PET, along with a low disease burden (1 or 2 lesions diagnosed with PSMA PET). As tissues can often become adhesive and fibrotic around blood vessels, and vessels themself may have been compromised due to previous surgery or radiation, the surgeon was prepared for the possibility of vascular injuries. The vascular clamps and the conversion set were readily available at the back table, for rapidly switching to an open surgical approach if needed. To minimize the risk of injury, the dissection process was conducted meticulously. Nevertheless, the surgical steps from an open approach could be easily adapted to the robotic approach.

All patients were informed about the experimental nature of the salvage surgery and signed a consent form for data recording and analysis. The surgical approach was deeply described prior to surgery with the patients.

### PSMA-RGS procedure

PSMA-RGS was performed as previously reported with either an open or a robotic minimal-invasive approach [6, 7, 9, 15].

Briefly, [^99m^Tc]Tc-PSMA-I&S was intravenously injected the day before surgery [15]. Prior to surgery, single-photon emission computed tomography/computed tomography (SPECT/CT) imaging cross-validated the PSMA PET findings, and assessed lesion uptake and tracer distribution [15, 16].

PSMA-RGS resection templates were based on the extend and location of PSMA-positive lesions that were assessed as suspicious for local recurrences and/or lymph node (LN) metastases on preoperative PSMA PET imaging. The extended salvage LN dissection (SLND) template of pelvic LNs included all stations distal of the aortic bifurcation (common iliac, external iliac, obturator and internal iliac nodes). Unilateral or bilateral templates were performed according to the surgeon’s discretion. For presacral/pararectal lesions, resection of the relevant region was conducted. In the case of retroperitoneal lesions, resection was performed according to a template typically used for testicular cancer, with a pelvic SLND at least on the corresponding side [9].

A DROP-IN gamma probe (Crystal Probe CXSSG603; Crystal Photonics, Berlin, Germany) was used for intraoperative in vivo assessment of radioactivity uptake [17]. Ex vivo gamma measurements immediately tested the successful dissection of positive lesions [6]. Histopathological examination was performed by dedicated and experienced uropathologists.

### Outcomes of interest

Oncological outcomes (rate of complete biochemical response [cBR], biochemical recurrence-free survival [BFS], rate of salvage therapy) were evaluated. cBR was defined as PSA <0.2 ng/ml 2–16 weeks after PSMA-RGS. BFS was defined as PSA <0.2 ng/ml without any further treatment. Patients were censored on the date of last evidence of freedom from BCR. The rate of salvage therapy was based on any further treatment following PSMA-RGS.

Intraoperative surgical outcomes (operative time [OT], estimated blood loss [EBL]) and postoperative complications according to Clavien-Dindo (CD) [18] classification were analyzed.

### Statistical analysis

Descriptive statistics included frequencies and proportions for categorical variables. Means, medians, and interquartile ranges (IQR) were reported for continuously coded variables. Wilcoxon rank sum test, Pearson’s Chi-squared test, and Fisher’s exact test were used to compare parameters between groups (robot assisted vs. open). The impact of surgical approach on BFS was analyzed by log-rank test and Kaplan–Meier plot. Univariable and multivariable models tested the influence of the open vs. robotic approach on surgical outcomes (OT and EBL).

Covariables for adjustment consisted of number of lesions removed during PSMA-RGS, PSA level at PSMA-RGS (all continuously coded), adjuvant radiation therapy after RP (no vs. yes), location of PSMA PET positive lesions prior to PSMA-RGS (lymph nodes vs. retrovesical/paravesical). For surgical outcomes, a subgroup analysis was performed that included only patients with unilateral positive pelvic lesions or retrovesical/paravesical lesions.

The R software environment for statistical computing and graphics (version 4.1.2) was used for all statistical analyses. All tests were two-sided with a level of significance set at *p* < 0.05.

## Results

Overall, 85 patients were included in the study, with 61 patients (72%) undergoing open PSMA-RGS and 24 patients (28%) receiving a robot-assisted approach (baseline patient’s characteristics are shown in supplementary (Table 1).

**Table 1:** Initial patients´ characteristics

Parameter	Open PSMA-RGS (*n* = 61)	Robot-assisted PSMA-RGS (*n* = 24)	*P* value
Year of initial RP, median (IQR)	2018 (2015, 2020)	2019 (2015, 2020)	0.42
PSA at RP (ng/ml), median (IQR)	9 (7, 12)	12 (8, 14)	0.42
pT stage at RP, *n* (%)			0.91
pT2	20 (33)	10 (42)	
pT3a	20 (33)	8 (33)	
pT3b	15 (25)	6 (25)	
NA	6 (9)	0 (0)	
Gleason grade group, *n* (%)			0.15
I–II	13 (22)	9 (38)	
III–V	42 (70)	15 (62)	
NA	6 (9)	0 (0)	
pN stage at RP, *n* (%)			0.81
pN0	42 (69)	17 (71)	
pN1	11 (18)	3 (12)	
pNx	8 (13)	4 (17)	
Lymph node yield at RP, median (IQR)	4 (7, 20)	10 (7, 15)	0.30
No. of positive lymph nodes at RP, *n* (%)			0.75
0	42 (70)	18 (75)	
1	7 (12)	1 (4)	
2	2 (3)	0 (0)	
≥ 3	2 (3)	1 (4)	
NA	8 (13)	4 (17)	
Surgical margin status, *n* (%)			0.06
R0	35 (57)	21 (88)	
R1	17 (28)	3 (12)	
Rx	2 (3)	0 (0)	
NA	7 (11)	0 (0)	
(RT after RP, *n* (%)			0.89
No RT post RP	32 (52)	13 (54)	
RT post RP	29 (48)	11 (46)	

*IQR* Interquartile range; *NA* not assigned; *PSA* prostate-specific antigen; *RP* radical prostatectomy; *RT* radiotherapy

No problems or complications occurred during intraoperative examination with the DROP-IN gamma probe. The median time interval between primary RP and PSMA-RGS was similar in both groups, with 42 months (interquartile range [IQR]: 22, 74) in the open group and 35 months (IQR: 13, 76) in the robotic group (Table 2).

**Table 2:** Characteristics of 85 patients at salvage PSMA-RGS

Parameter	Open PSMA-RGS (*n* = 61)	Robot-assisted PSMA-RGS (*n* = 24)	*P* value
Age at PSMA-RGS (year), median (IQR)	63 (60, 69)	64 (60, 67)	0.88
Time between RP and PSMA-RGS (mo), median (IQR)	42 (22, 74)	35 (13, 76)	0.61
PSA prior to PSMA-RGS (ng/ml), median (IQR)	0.58 (0.38, 0.87)	0.44 (0.31, 0.71)	0.14
No. of PSMA PET–avid lesions, *n* (%)			0.31
1	40 (66)	22 (92)	
2	11 (18)	2 (8)	
3	6 (9.5)	0 (0)	
≥4	4 (6.5)	0 (0)	
PSMA PET localization, *n* (%)			0.01
Pelvic unilateral	30 (49)	15 (62)	
Pelvic bilateral	4 (6.7)	0 (0)	
Pelvic plus presacral or retrovesical	2 (3.3)	0 (0)	
Presacral/pararectal	8 (13)	0 (0)	
Retroperitoneal	7 (11)	0 (0)	
Retroperitoneal plus other localization	3 (5)	0 (0)	
Retrovesical/paravesical	7 (11)	9 (38)	
Lymph node yield at SLND, median (IQR)	17 (11, 24)	12 (8, 21)	0.14
No. of pathologically positive lymph nodes, *n* (%)			
0	3 (5)	3 (12.5)	
1	24 (39)	12 (50)	
2	12 (20)	3 (12.5)	
3	8 (13)	2 (8.4)	
≥4	14 (23)	4 (16.6)	
Location of positive lesions, *n* (%)			0.09
Pelvic	28 (46)	12 (50)	
Pelvic plus presacral or retrovesical	1 (1.6)	0 (0)	
Presacral/pararectal	7 (11.5)	0 (0)	
Retroperitoneal	4 (6.6)	0 (0)	
Retroperitoneal plus other localization	11 (18)	2 (8.3)	
Retrovesical/paravesical	8 (13)	7 (29)	
Negative	2 (3.3)	3 (12.5)	

*IQR* interquartile range; *PET* positron emission tomography; *PSA* prostate-specific antigen; *PSMA* prostate-specific membrane antigen; *PSMA-RGS* PSMA-radioguided surgery

At the time of salvage surgery, the median PSA level was 0.58 ng/ml (IQR: 0.38, 0.87) in the open and 0.44 ng/ml (IQR: 0.31, 0.71) in the robotics, respectively (Table 2). Most of the patients who underwent robot-assisted PSMA-RGS had one positive lesion at PSMA PET prior surgery (92% vs. 66% in the open group). Of these, 15 (62%) patients had unilateral pelvic PCa recurrence and 9 (38%) had retrovesical/paravesical PCa recurrence at PSMA PET.

The pathological results confirmed 1 positive lesion in 24 (39%) patients in the open group and 12 (50%) patients in the robotic group, respectively. Negative pathological findings were reported in 3 (5%) cases among the open and 3 (12.5 %) patients in the robotics.

### Oncological outcomes

**Fig. 2:**
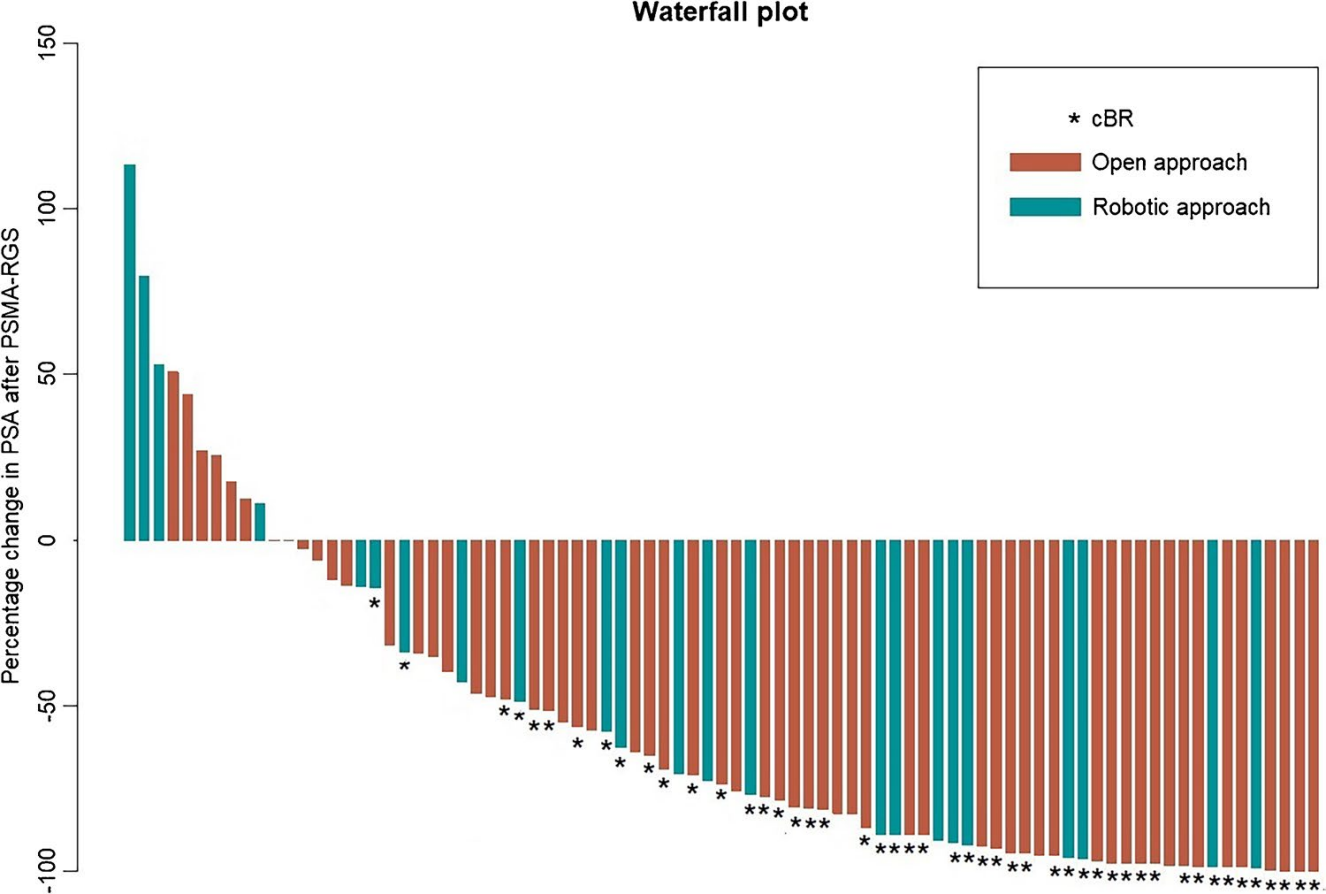
Waterfall plot graphically depicting the percentage of PSA change from before to after open or robotic prostate-specific membrane antigen–radioguided surgery (PSMA-RGS)

**Fig. 3:**
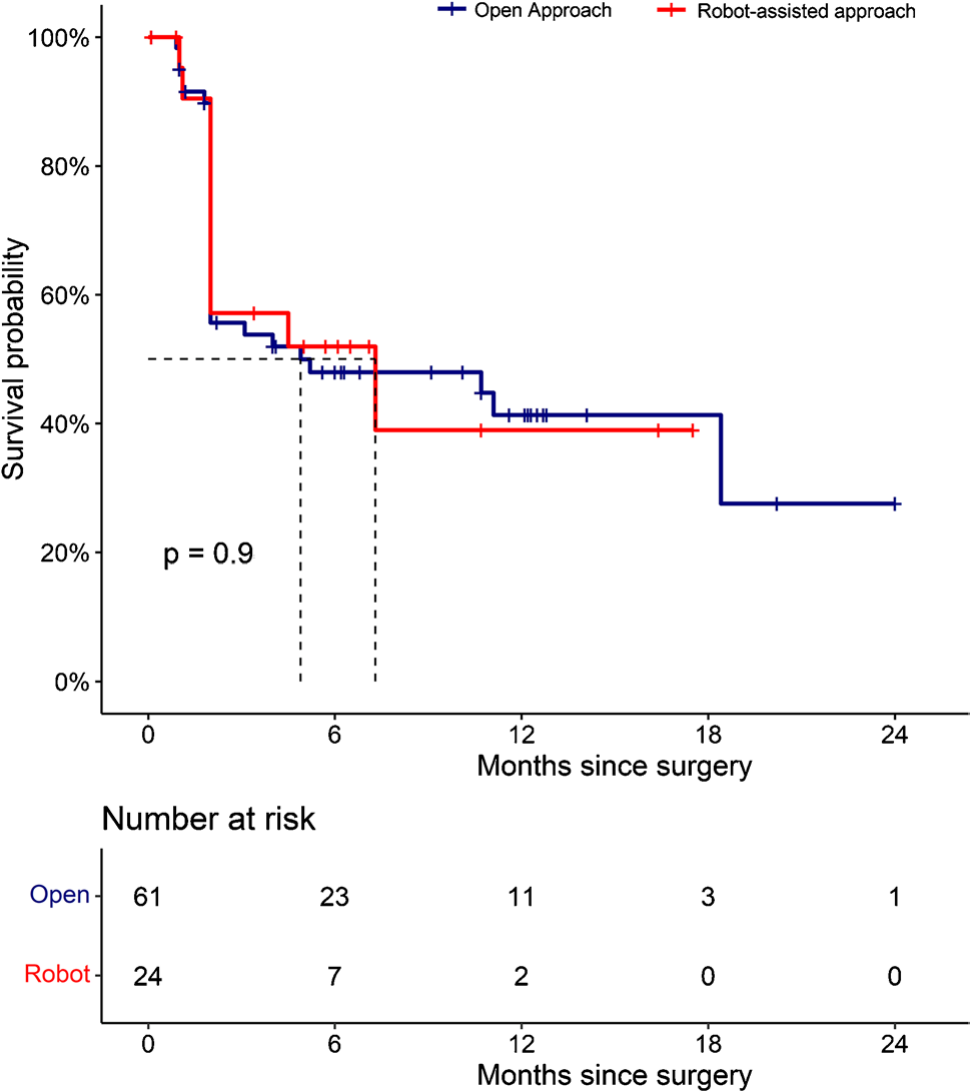
Kaplan–Meier analyses depicting biochemical recurrence–free survival rates in 85 patients treated with open (*n* = 61) or robot-assisted (*n* = 24) prostate-specific membrane antigen–radioguided surgery between January 2021 and December 2022 in one tertiary care center

The median follow-up was 10 months (IQR: 5, 13) for the open group and 6 months (IQR: 2, 14) for the robotic group, respectively (Table 3). cBR 2-16 weeks after PSMA-RGS was reported in 34 (57%) patients in the open group and 14 (61%) patients in the robotic group (Table 3; Fig. 2). 32 (52%) vs. 11 (46%) patients experienced BCR and 9 (15%) vs. 4 (17%) patients received further therapy during the follow-up in the open vs. robotic groups, respectively. According to the Kaplan–Meier analysis, the open and the robotic patients do not differ significantly in terms of BFS (12-month BFS: 41% (confidence interval (CI): 29–58%) vs. 39% (CI: 19–79%), *p* = 0.9, respectively) (Fig. 3).

**Table 3:** Oncological outcomes of 85 patients receiving open or robot-assisted PSMA-RGS

Parameter	Open PSMA-RGS (*n* = 61)	Robot-assisted PSMA-RGS (*n* = 24)	*P* value
PSA 2–16 week after RGS (ng/ml), *n* (%)	0.12 (0.05, 0.32)	0.09 (0.03, 0.27)	0.80
cBR, *n* (%)	34 (57)	14 (61)	0.70
BCR, *n* (%)	32 (52)	11 (46)	0.60
Salvage therapy, *n* (%)	9 (15)	4 (17)	0.90
Follow-up (months), median (IQR)	10 (5, 13)	6 (2, 14)	0.30

*cBR* Complete biochemical response; *BCR* biochemical recurrence; *IQR* interquartile range; *PET* positron emission tomography; *PSA* prostate-specific antigen; *PSMA* prostate-specific membrane antigen; *PSMA-RGS* PSMA-radioguided surgery

### Surgical outcomes

The robotic approach was associated with a lower median EBL (50 ml (IQR: 42–100) vs. 100 ml (IQR: 50–200) in ORP, *p* = 0.01) and a longer operative time (152 min [IQR: 105, 174] vs. 110 min [IQR: 95, 135], *p* = 0.02). No complications of CD III–V were reported in the robotic group (Table 4). No conversion to open surgery was necessary.

**Table 4:** Surgical outcomes of 85 patients receiving open or robot-assisted PSMA-RGS

Parameter	Open PSMA-RGS (*n* = 61)	Robot-assisted PSMA-RGS (*n* = 24)	*P* value
Operative time (min), median (IQR)	110 (95, 135)	152 (105, 174)	0.02
EBL (ml), median (IQR)	100 (50, 200)	50 (42, 100)	0.01
Postoperative complications (Clavien-Dindo), *n* (%)			0.50
I–II	18 (30)	6 (25)	
III–V	4 (6)	0 (0)	
None	39 (64)	18 (75)	

*IQR* Interquartile range; *EBL* estimated blood loss; *PSMA* prostate-specific membrane antigen; *PSMA-RGS* PSMA-radioguided surgery

Univariable linear regression analysis showed that the number of lesions retrieved (*β* = 5.3, 95% CI: 2.4, 8.2; *p* < 0.001) and post-RP radiation therapy (*β* = −89, 95% CI: −152, −25; *p* = 0.01) had a significant impact on EBL. After adjusting for these statistically significant independent predictors, the surgical approach did not significantly influence EBL (*β* = −40, 95% CI: −103, 22; *p* = 0.2). The univariable and multivariable regression models revealed a significant relationship between robotic approach and the increased OT (*β* = 25, 95% CI: 4.9, 45; *p* = 0.02; *β* = 28, 95% CI: 10, 46; *p* = 0.002, respectively) (Table 5).

**Table 5:** Univariable and multivariable linear regression models evaluating independent predictors for operative time and estimated blood loss in patients receiving open or robot-assisted PSMA-RGS

Variable	Operative time						Estimated blood loss					
	Univariable			Multivariable			Univariable			Multivariable		
	*β*	95% CI	*p* value	*β*	95% CI	*p* value	*β*	95% CI	*p* value	*β*	95% CI	*p* value
Surgical approach												
Open	Ref.			Ref.						Ref.		
Robot-assisted	25	4.9, 45	0.02	28	10, 46	0.002	−53	−126, 20	0.20	−40	−103, 22	0.20
PSA at PSMA-RGS (ng/ml)	−0.44	−7.1, 6.2	0.90				22	−0.63, 46	0.056			
Number of LNDs removed during PSMA-RGS *	1.6	0.81, 2.4	<0.001	1.6	0.83, 2.4	<0.001	5.3	2.4, 8.2	<0.001	4.3	1.4, 7	0.01
Location of PSMA PET positive lesions prior to PSMA-RGS												
Lymph nodes	Ref.						Ref.					
Retrovesical/paravesical	−25	−48, −1.5	0.04				−92	−175, −9.6	0.03			
Radiotherapy post-RP												
No	Ref.						Ref.			Ref.		
Yes	−15	−32, 2.2	0.09				−89	−152, −25	0.01	−58	−113, −3.6	0.04

*ADT* Androgen deprivation therapy; *CI* confidence interval; *PET* positron emission tomography; *PSA* prostate specific antigen; *PSMA* prostate-specific membrane antigen; *Ref.* reference; *RGS* radioguided surgery, *RP* radical prostatectomy

^*^Number of lymph nodes, not including local recurrence

Subgroup analysis in case of unilateral positive pelvic lesions or retrovesical/paravesical lesions detected by PSMA PET, along with a low disease burden (1 or 2 lesions diagnosed with PSMA PET) confirmed these findings (Table 6). Specifically, the surgical approach significantly affected operative time (*β* = 33, 95% CI: 12, 54; *p* = 0.003), while it did not significantly influence EBL (*β* = −26% CI: −86, 34; *p* = 0.4) (Table 6). Due to the low number of complications, no regression models were performed to analyze their association with the surgical approach.

**Table 6:** Subgroup analysis: univariable and multivariable linear regression models evaluating independent predictors for operative time and estimated blood loss in patients receiving open or robot-assisted PSMA-RGS in case of unilateral positive pelvic lesions or retrovesical/paravesical lesions detected by PSMA PET, along with a low disease burden (1 or 2 lesions diagnosed with PSMA PET)

Variable	Operative time						Estimated blood loss					
	Univariable			Multivariable			Univariable			Multivariable		
	*β*	95% CI	*p* value	*β*	95% CI	*p* value	*β*	95% CI	*p* value	*β*	95% CI	*p* value
Surgical approach												
Open	Ref.			Ref.						Ref.		
Robot-assisted	26	3.6, 49	0.03	33	12,54	0.003	−46	−113, 21	0.2	−26	−86, 34	0.4
PSA at PSMA-RGS (ng/ml)	−6.6	−18, 4.8	0.02				37	6.1, 69	0.02			
Number of lesions removed during PSMA-RGS	1.7	0.61, 2.7	0.003	1.9	0.93, 2.9	<0.001				6.1	3.2, 9	<0.001
Location of PSMA PET positive lesions prior to PSMA-RGS												
Lymph nodes	Ref.						Ref.					
Retrovesical/paravesical	−30	−55, −4.9	0.02				−88	−144, −18	0.01			
Radiotherapy post-RP												
No	Ref.						Ref.					
Yes	−22	−40, −3.9	0.06				−81	−144, −18	0.01			

*CI* Confidence interval; *PET* positron emission tomography; *PSA* prostate specific antigen; *PSMA* prostate-specific membrane antigen; *Ref.* reference; *RGS* radioguided surgery, *RP* radical prostatectomy

## Discussion

PSMA-RGS has become a promising experimental technique in the management of PCa recurrence [1, 13, 14] and is currently performed predominantly with an open approach. Literature on the minimally invasive approach in salvage PSMA-RGS is decidedly scarse [19].

We performed a retrospective comparative evaluation between patients submitted to open or robot-assisted PSMA-RGS for recurrent PCa and our analysis yielded valuable results.

First, we found that the oncological outcomes of the robotic approach are comparable to those of open surgery. Previous literature addressing this endpoint is limited. Only one prospective feasibility study reported a 29% cBR rate in patients who had previously undergone prior radical prostatectomy (*n*=14) [13]. Our results are consistent with the largest series of salvage PSMA-RGS, including both robotic and open approaches (*n*=364) that reported a 2-year BFS of 32% [9]. Despite the short follow-up and limited sample size, our results are of great importance as they reassure clinicians and patients that opting for the robotic technique does not compromise the efficacy of the surgical intervention. Therefore, the debate on this topic should not focus primarily on the surgical approach but, more importantly, on the selection of patients for PSMA-RGS itself. Salvage PSMA-RGS appears to be a viable long-term treatment option for a subgroup of patients, particularly those with low PSA levels and a low number of positive lesions on PSMA-PET [9, 20]. Nevertheless, the lack of specific biomarkers and large prospective trials makes the selection of the most appropriate candidate challenging.

Second, our study compared surgical outcomes between the two groups, which, to our knowledge, isfirst available comparative analysis on this innovative topic in the literature. We found that the robotic approach increased the OT compared with the open surgery. While this finding may suggest a potential drawback, it is worth noting that the switch from the open PSMA-RGS to the minimally invasive approach could justify the longer operative time despite the surgeon’s extensive experience. Caution should be taken when interpreting this result, and we believe that the robotic approach has the potential to achieve comparable OT with increasing experience and proficiency.

Furthermore, our analysis revealed that the robotic approach did not significantly reduce intraoperative EBL. The initial experience and lower confidence in minimally invasive surgery may contribute to the potential benefits of robotics in EBL.

To address potential selection bias of patients undergoing robotic surgery, a subgroup analysis including patients with unilateral positive pelvic lesions or retrovesical/paravesical lesions detected by PSMA PET, and low disease burden (1 or 2 lesions diagnosed with PSMA PET) was conducted to reassess the surgical outcomes. The results of this secondary analysis confirmed the initial findings.

The robotic approach was usually recommended for a specific group of patients who had unilateral positive pelvic lesions or retrovesical/paravesical lesions with a low disease burden. On the other hand, patients with a higher disease burden or more challenging cases, such as those with previous abdominal surgeries or PCa recurrences in anatomically difficult areas such as pararectal lesions, were scheduled for open surgery resulting in selection bias. Moreover, the prior surgical approach to RP was considered when counseling patients.

Nevertheless, the robot-assisted PSMA-RGS offered enhanced accessibility for addressing local recurrences, as it allowed better visualization and maneuverability in the paravesical/retrovesical region. This more comfortable access may enable the surgeon to target and remove the recurrent tumor more effectively, while minimizing damage to the surrounding tissues.

The advantages of the minimally invasive approach may also include faster postoperative recovery and greater patient comfort. In the case of robotic PSMA-RGS after minimally invasive RP, the previous small incisions were used, which may reduce postoperative discomfort. All of these points can contribute to an overall improvement in patients’ quality of life as they can experience less pain, have shorter hospital stays, and can return to their daily activities more quickly.

There are limited data in the existing literature on the potential benefits of a minimally invasive approach for salvage surgery on PCa local recurrences. A case report describing two cases of robot-assisted resection of local recurrence after RP is available highlighting the potential advantages of a minimally invasive approach for salvage surgery on local recurrences [21].

Most of the studies available in the literature used the open approach for salvage lymph node dissection in patients with nodal recurrence after RP [22]. The surgical outcomes of robot-assisted salvage nodal dissection without tracer guidance for lymph node recurrence after RP was evaluated in small either prospective [23] or retrospective series [23–27]. All of these studies supported the feasibility and safety of the minimally invasive approach, but the lack of high-level-evidence analyses did not allow reliable comparison between surgical approaches.

Lastly, evaluation of complications revealed an overall moderate rate in both groups. Notably, no severe complications (CD III–V) were recorded in the robotic patients, further supporting the minimally invasive approach. Complications following SLND and metastasis-directed treatments should be considered when counseling patients. Up to 20% of patients may suffer from at least CD III complications such as lymphocele requiring drainage, ureteral stricture or sepsis, and pulmonary embolism [28]. Nevertheless, several studies suggested that performing salvage surgery using a minimally invasive approach may reduce morbidity [29]. Abreu et al. demonstrated reduced morbidity even with an extended template after minimally invasive SLND [23]. Linxweiler et al. analyzed 68 robot-assisted SLND and no complications CD > III were reported [25].

The implementation of PSMA-RGS can potentially enhance the benefits of a minimally invasive approach for salvage surgery allowing for more targeted surgical excision. By acquiring proficiency through experience and thorough long-term follow-up, it will be possible to collect more comprehensive data on the potential benefits and efficacy of minimally invasive PSMA-RGS for salvage surgery in PCa recurrence.

Some limitations should be acknowledged. First, the sample size was relatively small and unbalanced, which may have limited the generalizability of our findings and may have affected regression models with overfitting. Second, long-term oncological results were not available due to the limited follow-up. Furthermore, the retrospective nature of the study introduces inherent limitations, including possible selection bias. Additionally, the learning curve associated with adopting the robotic approach may have influenced our results, including OT and EBL. The robotic approach was recommended for a subgroup of patients leading to selection bias. Therefore, caution should be taken when analyzing oncological and surgical outcomes of patients with a different pattern of recurrence.

Considering these limitations, further studies are needed to assess oncological and surgical impact of robot-assisted PSMA-RGS. Nevertheless, our preliminary study provides valuable insights into the surgical and short-term oncological outcomes associated with robot-assisted PSMA-RGS compared with open surgery, and highlighted its safety and feasibility.

## Conclusion

Both, the robot-assisted and the open approach for PSMA-RGS in recurrent PCa patients showed comparable oncological outcomes. The robot-assisted PSMA-RGS was associated with longer OT and equal EBL compared with open surgery. The minimally invasive approach appeared to be safe, as no safety signals were reported. Additionally, it has the potential to reduce morbidity associated with traditional open surgery.

## Data Availability

The datasets analyzed during the current study are available from the corresponding author on reasonable request.
